# Metformin Combining PD-1 Inhibitor Enhanced Anti-Tumor Efficacy in *STK11* Mutant Lung Cancer Through AXIN-1-Dependent Inhibition of STING Ubiquitination

**DOI:** 10.3389/fmolb.2022.780200

**Published:** 2022-02-23

**Authors:** Zhiguo Wang, Conghua Lu, Kejun Zhang, Caiyu Lin, Fang Wu, Xiaolin Tang, Di Wu, Yuanyao Dou, Rui Han, Yubo Wang, Chao Hou, Qin Ouyang, Mingxia Feng, Yong He, Li Li

**Affiliations:** ^1^ Department of Respiratory Disease, Daping Hospital, Third Military Medical University (Army Medical University), Chongqing, China; ^2^ Department of Outpatients, Daping Hospital, Third Military Medical University (Army Medical University), Chongqing, China; ^3^ Department of Oncology, Hunan Key Laboratory of Tumor Models and Individualized Medicine, Hunan Key Laboratory of Early Diagnosis and Precision Therapy in Lung Cancer, The Second Xiangya Hospital, Central South University, Changsha, China; ^4^ School of Pharmacy, Third Military Medical University (Army Medical University), Chongqing, China

**Keywords:** metformin, lung cancer, STING, AXIN-1, immunotherapy, *STK11*

## Abstract

**Background:** Non-small-cell lung cancer (NSCLC) with *STK11* mutation showed primary resistance to immune checkpoint inhibitors (ICIs). The glucose-lowering drug metformin exerted anti-cancer effect and enhanced efficacy of chemotherapy in NSCLC with *KRAS/STK11* co-mutation, yet it is unknown whether metformin may enhance ICI efficacy in *STK11* mutant NSCLC.

**Methods:** We studied the impact of metformin on ICI efficacy in *STK11* mutant NSCLC *in vitro* and *in vivo* using colony formation assay, cell viability assay, Ki67 staining, ELISA, CRISPR/Cas9-mediated knockout, and animal experiments.

**Results:** Through colony formation assay, Ki67 incorporation assay, and CCK-8 assay, we found that metformin significantly enhanced the killing of H460 cells and A549 cells by T cells. In NOD-SCID xenografts, metformin in combination with PD-1 inhibitor pembrolizumab effectively decreased tumor growth and increased infiltration of CD8^+^ T cells. Metformin enhanced stabilization of STING and activation of its downstream signaling pathway. siRNA-mediated knockdown of *STING* abolished the effect of metformin on T cell-mediated killing of tumor cells. Next, we found that CRISPR/Cas9-mediated knockout of the scaffold protein AXIN-1 abolished the effect of metformin on T cell-mediated killing and STING stabilization. Immunoprecipitation and confocal macroscopy revealed that metformin enhanced the interaction and colocalization between AXIN-1 and STING. Protein-protein interaction modeling indicated that AXIN-1 may directly bind to STING at its K150 site. Next, we found that metformin decreased K48-linked ubiquitination of STING and inhibited the interaction of E3-ligand RNF5 and STING. Moreover, in *AXIN-1* ^*−/−*^ H460 cells, metformin failed to alter the interaction of RNF5 and STING.

**Conclusion:** Metformin combining PD-1 inhibitor enhanced anti-tumor efficacy in *STK11* mutant lung cancer through inhibition of RNF5-mediated K48-linked ubiquitination of STING, which was dependent on AXIN-1.

## Introduction

Lung cancer is the leading cause of cancer-related deaths worldwide, despite the mortality has fallen continuously due to improved treatment (Siegel et al., 2021). Immune checkpoint inhibitors (ICIs), including PD-1/PD-L1 inhibitors, have produced remarkably durable responses in advanced non-small-cell lung cancer (NSCLC) (Qu et al., 2021). However, only a minority of patients achieve durable benefit from ICIs and the landscape of primary resistance to PD-1 blockade is largely unknown. Mutation of *STK11* (Liver Kinase B1-LKB1), a tumor suppressor gene which encodes an evolutionary conserved serine/threonine kinase (Sanchez-Cespedes et al., 2002), has been suggested to be a potential driver of primary resistance to PD-1 blockade (Jure-Kunkel et al., 2018; Skoulidis et al., 2018). Mechanistically, *STK11* mutation leads to LKB1 loss, which then results in the suppression of stimulator of interferon genes (STING) (Kitajima et al., 2019), whose activation is critical for anti-cancer immune response (Su et al., 2019; Zhu et al., 2019). Therefore, activation of STING in *STK11* mutant cancer is a promising approach to convert an immune-resistant, noninflamed tumor into an immune-sensitive, inflamed tumor.

Mounting evidence has suggested that the anti-diabetes drug metformin exerted anti-cancer effect in various cancer types, including lung, prostate, and colon (Kirtonia et al., 2021). Previous studies have found that *STK11* mutation can lead to increased sensitivity of cells to metformin or other inhibitors of mitochondrial respiration by restraining their ability to upregulate glucose uptake and glycolysis (Shackelford et al., 2013; Parker et al., 2017). Indeed, metformin enhanced cisplatin-induced apoptosis in *KRAS/STK11* co-mutated NSCLC (Moro et al., 2018). More recently, metformin can induce STING expression in pancreatic cancer and activate the STING/IRF3/IFN-β pathway by inhibiting AKT signaling in pancreatic ductal adenocarcinoma (Ren et al., 2020). However, it is unknown whether metformin may enhance the efficacy of immunotherapy in *STK11* mutant lung cancer. Therefore, we aim to study whether metformin can enhance T cell-mediated killing of *STK11* mutant lung cancer and the underlying mechanisms.

## Materials and Methods

### Cell Culture and Reagents

The human lung cancer cell lines H460, A549, and 293T cell were purchased from the American Type Culture Collection (ATCC). Human peripheral blood mononuclear cells (PBMC) and human peripheral blood T cells were both from SAILYBIO (Shanghai, China). Cells were cultured in RPMI-1640 (Hyclone) supplemented with 10% fetal bovine serum (FBS, Gibco) and 1% penicillin/streptomycin at 37°C in a humidified 5% CO_2_ atmosphere. Metformin and MG132 were from Selleck (TX, United States), and pembrolizumab were from MSD (NJ, United States). Antibodies against STING, TBK1, p-TBK1, IRF3, p-IRF3, CD8 and β-tubulin were purchased from Cell Signaling Technology (MA, United States), and those against RNF5, RNF26, TRIM32, and TRIM56 were from Abcam (Cambridge, United Kingdom), and anti-Flag tag and anti-His tag were from Bioss (Beijing, China).

### Generation of Activated T Cells

Activated T cells were acquired as previously reported (Li et al., 2016). Briefly, human peripheral blood T cells were cultured in ImmunoCult-XF T cell expansion medium with ImmunoCult Human CD3/CD28/CD2 T cell activator (both from STEMCELL Technologies, Vancouver, CA, United States) and IL-2 (10 ng/ml; PeproTech, NJ, United States) for 1 week according to the manufacturer’s protocol. All experiments were performed in DMEM/F12 medium with anti-CD3 antibody (100 ng/ml; eBioscience, Thermo Scientific, MA, United States) and IL-2 (10 ng/ml).

### Colony Formation Assay

Cancer cells were incubated with activated T cells for 48 h with or without metformin. The ratios between cancer cells and activated T cells were set as 1:1. T cells and cell debris were removed by PBS wash, and cancer cells were left to grow for 2 weeks and then the colonies were subjected to crystal violet staining.

### Cell Viability Assay

Cell viability was determined by cell counting kit-8 (CCK8; MedChemExpress, NJ, United States) according to the operation manual. Briefly, cells were seeded in a 96-well plate at a density of 3 × 10^3^ per well and cultured overnight. On the next day the medium was refreshed with the indicated doses of drug-containing medium and cultured for another 48 h. Then the medium was refreshed and absorbances were measured at 450 nm on a Sunrise R microplate reader (Thermo Fisher Scientific, Germany).

### Ki67 Staining

Cell proliferation was assessed by the Ki67 incorporation assay with a Ki67 labeling and detection kit (Boster, Wuhan, China). Briefly, cells were seeded in six-well plates (3 × 10^5^) and treated as indicated for 48 h. Then cells were fixed and incubated overnight with Ki67 (1:200 dilution). Cells were counterstained with 4′, 6-diamidino-2-phenylindole (DAPI) for 15 min and observed under a fluorescence microscope.

### Enzyme-Linked Immunosorbent Assay

IFN-γ levels were measured by ELISA assay (Solarbio, Beijing, China). Briefly, the conditioned medium from human peripheral blood T cells of different groups was collected and assayed according to the manufacturer’s instructions. Values represent the average of three replicates from at least three independent experiments.

### siRNA Transfection

Small interfering RNAs (siRNAs) were synthesized by RiboBio Co., Ltd. (Guangzhou, China). For the evaluation of efficacy, H460 cells cultured in 6-well plates were transfected with either 100 pmol siRNA (sequences: CTG​GCA​TGG​TCA​TAT​TAC​A; ACA​GCA​ACA​GCA​TCT​ATG​A; GGA​TTC​GAA​CTT​ACA​ATC​A) or negative control siRNA (siNC) using Lipofectamine RNAiMAX (Thermo Fisher Scientific, MA, United States), following the manufacturer’s instructions. At 72 h post-transfection, knockdown efficiency was determined by examining endogenous protein expression by Western blot.

### Establishment of PBMCs-CDX Mouse Model (Cell-Derived Xenograft)

Animal experiments were approved by the ethics committee on animal experimentation of the Army Medical University. To establish PBMCs-CDX mouse model (Lin et al., 2018), H460 cells (5 × 10^5^) were injected into the hind flanks of 6-8-week-old female NOD-SCID BALB/c mice. The mice were subjected to tumor growth monitoring, and tumor volumes were calculated from caliper measurements using the following formula: (length × width^2^)/2. When tumors reached 80–100 mm^3^ in volume, 5 × 10^6^ human PBMCs were intravenously transplanted. PD-1 inhibitor (Pembrolizumab, 25 mg/kg) was injected every 3 days intraperitoneally. Metformin (1 mg/ml) was dissolved in drinking water and given to mice orally. Tumor growth was monitored every 3 days. Cohorts were sacrificed when control mice tumors reached 15 mm in any direction measured. The levels of serum Aspartate aminotransferase (AST), Alanine aminotransferase (ALT), Urea and Creatinine were analyzed using the Beckman Coulter AU5821 auto-Chemistry System (CA, United States). Tumors were harvested, fixed with 4% paraformaldehyde, and embedded in paraffin. The infiltration of CD8^+^ T cells were stained with immunohistochemistry (IHC) and the expression of STING was determined by immunofluorescence staining according to the manufacturers’ instructions.

### CRISPR/Cas9-Mediated Knockout of *AXIN-1* and Construction of AXIN1 Expression

To generate *AXIN-1* ^−/−^ cell lines, CRISPR/Cas9 gene editing technology was utilized as reported (Moser et al., 2021) Two independent sgRNAs targeting *AXIN-1* were designed using the online tool from Zhang lab (http://crispr.mit.edu; sg *AXIN-1* -1: TTC​TGA​GGG​AGT​CTT​CCG​GG; sg *AXIN-1* -2: GGA​TCC​GTA​AGC​AGC​ACC​GC). The sgRNAs were incorporated into the plentiCRISPR v2-Blast (Plasmid #83480, Addgene) construct to generate lentivirus. After transfection, single-cell isolation, and expansion, *AXIN-1* ^−/−^ and control cell lines were obtained and confirmed using both genome sequencing and Western blot analysis. To generate cell lines overexpressing AXIN1, the human *AXIN-1* cDNA sequence (Genebank accession number: NM_003502) was searched for suitable target sequences. LentiCRISPRv2-AXIN1-2 was designed and generated by Sino Biological (Suzhou, China). The transfection efficiency was determined by examining endogenous protein expression by Western blot.

### Western Blot and Immunoprecipitation

Western blot was performed as previously described (Li et al., 2019a). Briefly, cells were harvested from 6-well plates after washing with PBS and lysed for 30 min at 4°C in RIPA lysis buffer (Sigma-Aldrich, France) added with 1% protease and phosphatase inhibitors. The quantitative analysis of protein was determined by the BCA protein assay kit after centrifugation at 12,000 *g* for 20 min at 4°C. Equal amounts of protein were loaded to SDS-PAGE gels and then transferred to polyvinylidene difluoride (PVDF) membranes (Millipore, German), which were then blocked with 5% non-fat milk in TBST for at least 1 h at room temperature and incubated with primary antibodies overnight at 4°C. Then the membranes were washed with TBST, and incubated with horseradish peroxidase-conjugated goat anti-rabbit or anti-mouse IgG (Cell Signaling Technology, USA) for 1 h. After that, the membranes were imaged with ChemiDoc Touch System (Bio-Rad, USA).

The immunoprecipitation experiment was performed according to the manufacturer’s instructions. Cell lysates were incubated with respective antibodies (1:50) overnight, followed by adding the protein A/G beads and incubated overnight at 4°C. Then, the immunoprecipitates were washed 3 times and levels of total proteins were analyzed by western blot as described.

### IP-Mediated Endogenous Ubiquitination Assay

To detect STING ubiquitination, H460 or 293T cells were transfected with plasmids containing His-Ubi and Flag-STING (SinoBiological, Beijing, China) according to the manufacturer’s instructions. Cells were cultured with metformin for 36 h and further incubated with MG132 (20 μM, Selleck) for another 12 h and then lysed. The cell lysates were then boiled for 10 min after adding 1% SDS and diluted to 0.1% SDS with lysis buffer. Protein concentrations of the extracts were measured, and equal amounts of extracts were used for immunoprecipitation of target protein.

### Homology Modeling of AXIN-1 and STING and Protein-Protein Docking Prediction

Due to the full-length structure of AXIN-1 and STING was not available, a homology model was generated. The amino acid sequence was downloaded from Uniprot protein database (https://www.uniprot.org/uniprot/O15169; https://www.uniprot.org/uniprot/Q86WV6). Homology modeling of Axin-1 and Sting was constructed through I-TASSER server (Yang and Zhang, 2015). Parameters for modeling were set as default values. The docking study was performed using Hex 8.0.0 software (Li et al., 2019b). The binding interactions were generated using PyMOL. Parameters for docking were set as default values.

### Statistical Analysis

All data are expressed as mean ± SEM and the statistical analysis was performed by GraphPad Prism 8 for Windows, GraphPad Software, San Diego, CA, United States, www.graphpad.com. Differences between two groups were analyzed by Student’s t test. A *p* value <0.05 was considered statistically significant.

## Results

### Metformin Enhanced T Cell-Mediated killing of *STK11* Mutant Lung Cancer Cells *In Vitro*

**FIGURE 1:**
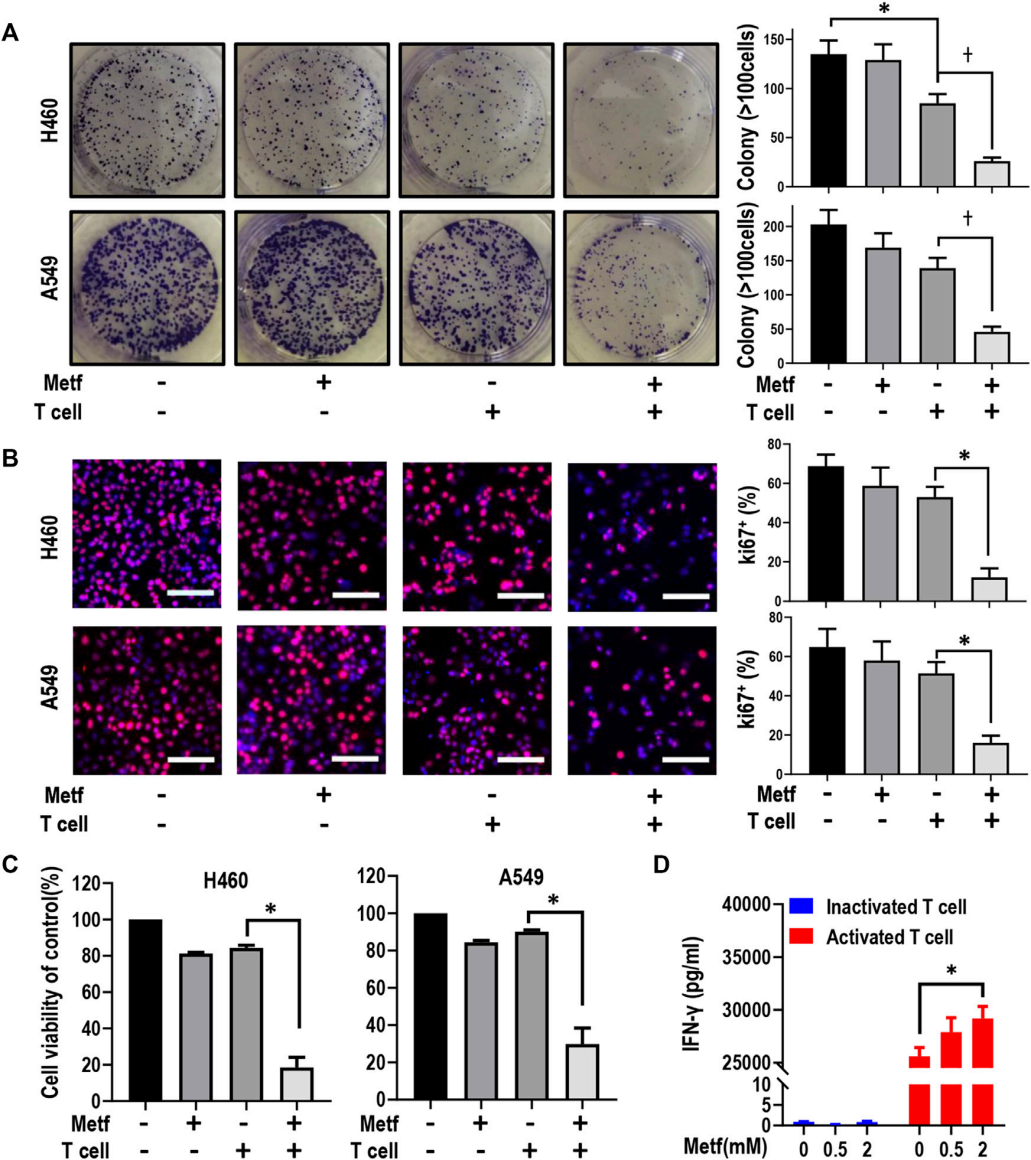
Metformin enhanced T cell-mediated killing of lung cancer H460 and A549 cells. **(A)**, H460 cells and A549 cells were co-cultured with activated T cells (cancer cells to T cells ratio, 1:1) for 48 h with or without metformin (0.5 mM). Cell colonies were visualized by crystal violet staining. *p < 0.05; ^†^ *p* < 0.01. **(B)**, Ki67 incorporation assay on H460 cells and A549 cells treated as indicated. Activated T cells (1:1 ratio to cancer cells) or metformin (0.5 mM) were added to the culture medium for 48 h. Cells were then counterstained with DAPI. Data represent mean ± SEM. *p < 0.01. Scale bars: 100 μm. **(C)**, Cell viability CCK-8 assay for cells treated with metformin (0.5 mM), or activated T cells (1:1 ratio to cancer cells), or the combination. Data are shown as mean ± SEM of triplicate determinations. *p < 0.01. **(D)**, ELISA analysis of the protein expression level of IFN-γ in inactivated or activated T cells with the treatment with metformin (0 mM, 0.5 mM, or 2 mM, respectively) for 24 h. Data presented as the mean ± SEM of three independent experiments. *p < 0.05.

We first asked whether metformin can enhance T-cell mediated killing of lung cancer cells with *STK11* mutation. Colony formation assay was performed in H460 cells and A549 cells. As shown in Figure 1A and Supplementary Figure S1, activated T cells (1:1 ratio to cancer cells) or metformin (0.5 mM) alone showed little effect on colony formation of both cell lines, while the combination significantly decreased cancer cell growth. Conversely, inactivated T cells alone or in combination with metformin had little effect on tumor cell proliferation (Supplementary Figure S2). Next, the Ki67 incorporation assay and CCK-8 assay was applied to measure cell proliferation. Similarly, activated T cells or metformin alone failed to inhibit cell proliferation, while the combination significantly decreased the percentage of Ki67-positive cells (Figure 1B) and decreased cell viability (Figure 1C). As previously reported, the tumor cell killing by cytotoxic T cells was in large part mediated by the pro-apoptotic effects of IFN-γ (Mezzadra et al., 2019). We then performed ELISA assay and found that metformin treatment in inactivated T cells had little effect on interferon (IFN)-γ secretion, while it increased the release of IFN-γ in activated T cells in a dose-dependent manner (Figure 1D). Taken together, these results suggest that metformin enhanced T cell-mediated killing of *STK11* mutant lung cancer cells *in vitro*.

### Metformin Enhanced the Anti-Tumor Efficacy With PD-1 Inhibitor *In Vitro* and *In Vivo*

We next asked whether metformin can enhance the anti-tumor efficacy with PD-1 inhibitor. We performed Ki67 incorporation assay *in vitro*. As shown in Supplementary Figure S3, in the presence of activated T cells, addition of pembrolizumab showed little effect on cancer cell growth, while addition of metformin further decreased the proliferation of both H460 cells and A549 cells. More importantly, the combination of metformin with pembrolizumab significantly decreased the percentage of Ki67-positive cells to a much greater extent, which indicates that metformin enhanced anti-tumor efficacy with PD-1 inhibitor *in vitro.*

**FIGURE 2:**
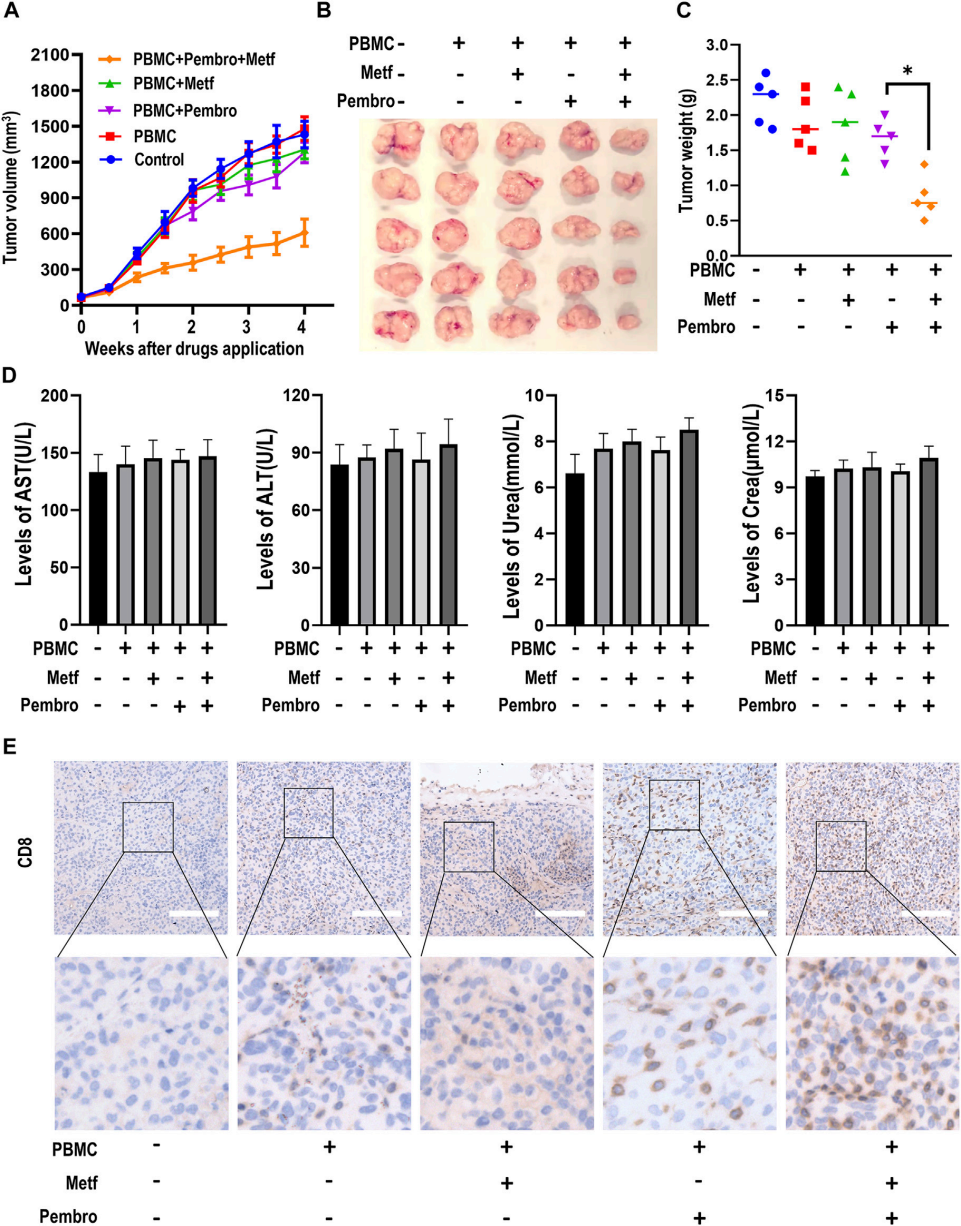
Metformin enhanced the efficacy of PD-1 inhibitor in PBMCs-CDX mouse model. **(A)**, Tumor growth curve of H460-derived xenograft mouse models treated as indicated. Tumor volume was shown as mean ± SEM (*n* = 5). *, *p* < 0.01 compared with the pembrolizumab group. **(B)**, Macroscopic appearance of tumors after drug application for 4 weeks **(C)**, Tumor weight (g) of each mouse was shown. *, *p* < 0.01. **(D)**, The levels of serum AST, ALT, Urea and Creatinine in mice serum were presented as mean ± SEM (*n* = 5). **(E)**, Immunohistochemistry analysis of CD8 in tumor sections from different groups. Representative images were shown. Scale bars: 200 μm. AST, Aspartate aminotransferase; ALT, Alanine aminotransferase; Crea, Creatinine.

We next examined the effects of combination of metformin and PD-1 inhibitor on tumor growth in a PBMCs-CDX mouse model. Intravenous injection of PBMCs alone had little effect on tumor growth. PBMCs plus metformin or pembrolizumab also did not inhibit the growth of tumors. In contrast, the combination of pembrolizumab and metformin resulted in a significant reduction of tumor volume (Figures 2A,B). Next, the tumor weight of each group was calculated and mapped in a scatter plot, which confirmed that tumors from the combination group had the lowest weight (Figure 2C). Meanwhile, there is no significant difference of mice body weight, or the level of AST, ALT, urea, and creatinine among groups (Figure 2D; Supplementary Figure S4). We next investigated the infiltration of CD8^+^ T cells in tumor microenvironment among groups by IHC. Metformin or pembrolizumab alone led to a slight increase of CD8^+^ T cell numbers, while the combination resulted in a remarkable CD8^+^ T cell infiltration (Figure 2E; Supplementary Figure S5). Taken together, these findings indicate that combined treatment of metformin and PD-1 inhibitor can suppress tumor growth and promote T-cell infiltration *in vivo*.

### Metformin Enhanced T Cell-Mediated Killing Through Stabilization of STING

**FIGURE 3:**
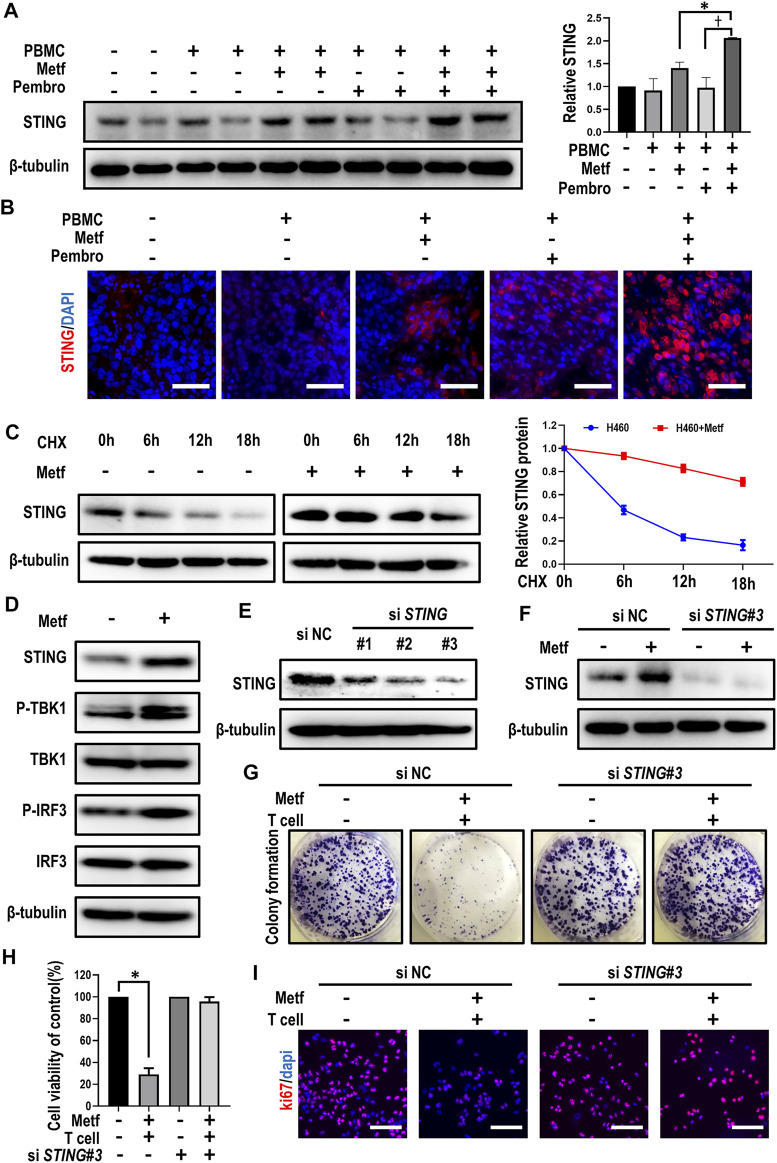
Metformin enhanced T cell-mediated killing through stabilization of STING. **(A)**, Western blot analysis of STING from tumor sections of each group and expression levels were presented. *p < 0.05; ^†^ *p* < 0.01. **(B)**, Immunofluorescence staining of STING from tumor sections of different groups as indicated. Scale bars: 30 μm. **(C)**, H460 cells were treated with 10 μM CHX at indicated intervals in the presence of metformin or not, and the level of STING was quantified using ImageJ software. **(D)**, Western blot analysis of indicated proteins in H460 cells with metformin treatment. **(E)**, Western blot showing the expression levels of STING in H460 cells after transfection with control or *STING* siRNAs, respectively. **(F)**, Western blot analysis of STING expression under metformin treatment after siRNA-mediated knockdown of STING. **(G)**, H460 cells transfected with control or *STING* siRNAs were co-cultured with activated T cells (cancer cells to T cells ratio, 1:1) for 48 h with or without metformin and then subjected to crystal violet staining. **(H)**, Cell viability CCK-8 assay for cells treated as indicated. Data are shown as mean ± SEM of triplicate determinations. *p < 0.01. **(I)**, Ki67 incorporation assay on H460 cells treated as indicated. Cells were counterstained with DAPI.

Previously, inactivation of STING was suggested to be responsible for resistance to immunotherapy in *STK11* mutant cells (Kitajima et al., 2019). We then asked whether activation of STING signaling was involved in enhanced T cell-mediated killing of cancer cells by metformin. Western blot analysis and immunofluorescence staining of tumor tissues from PBMCs-CDX mouse model described earlier showed increased expression of STING in the combination group (Figures 3A,B). Next, we determined whether metformin affects STING degradation in order to clarify the mechanism by which metformin modulates STING levels. In the presence of the protein synthesis inhibitor cycloheximide (CHX), metformin substantially slowed the degradation of STING in comparison with control (Figure 3C), suggesting that STING is stabilized in metformin-treated H460 cells. Previously, cGAS-STING-TBK1 signaling pathway was reported to play a crucial role in the anti-tumor immunity (Ding et al., 2020; Ren et al., 2020). Then, western blot results showed that expression of STING and downstream p-TBK1 and p-IRF3 were all increased by metformin treatment (Figure 3D), which suggests that metformin can activate STING signaling pathway.

We then asked whether STING activation was required for metformin-enhanced T cell-mediated killing of cancer cells. After small interfering (si) RNA-mediated knockdown of *STING*, metformin failed to increase STING expression in H460 cells (Figures 3E,F). Moreover, in siNC group, metformin together with activated T cells significantly decreased colony numbers and sizes of tumor cells, while the knockdown of *STING* abolished this effect (Figure 3G; Supplementary Figure S6A). Similarly, the combination of metformin and activated T cells decreased cell viability and inhibited cell proliferation in siNC group, yet this effect was abrogated after knockdown of *STING* (Figures 3H,I; Supplementary Figure S6B). Overall, these findings suggested that enhancement of T cell-mediated killing of cancer cells by metformin is affected by expression of STING.

### AXIN-1 Was Required for Metformin to Stabilize STING

**FIGURE 4:**
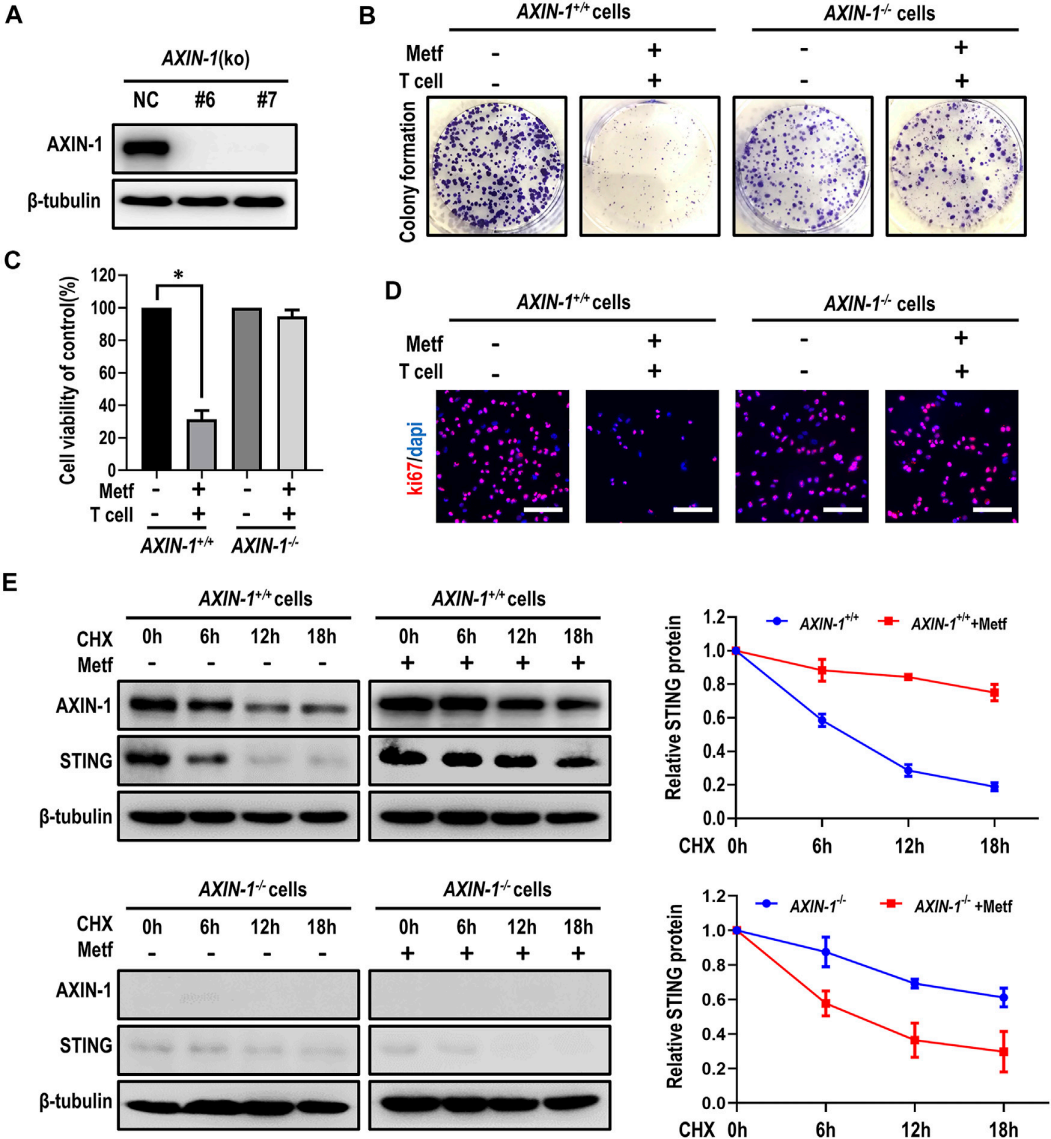
AXIN-1 was required for metformin to stabilize STING. **(A)**, Cell lysates from AXIN-1 knockout clones of H460 cells by CRISPR/CAS9 (KO-6 and KO-7) were subjected to western blot. **(B)**, *AXIN-1* ^*+/+*^cells and *AXIN-1* ^*−/−*^ cells co-cultured with activated T cells (cancer cells to T cells ratio, 1:1) for 48 h with or without metformin were subjected to crystal violet staining. **(C)**, Cell viability CCK-8 assay for *AXIN-1* ^*+/+*^cells and *AXIN-1* ^*−/−*^ cells treated as indicated. Data are shown as mean ± SEM. *p < 0.01. **(D)**, Ki67 incorporation assay on *AXIN-1* ^*+/+*^cells and *AXIN-1* ^*−/−*^ cells treated as indicated. Cells were counterstained with DAPI. **(E)**, *AXIN-1* ^*+/+*^cells and *AXIN-1* ^*−/−*^ cells were treated with 10 μM CHX at indicated intervals in the presence of metformin or not. The intensity of STING protein was quantified using ImageJ software.

It has been reported that the scaffold protein AXIN-1, which tether LKB1 to AMPK, plays an essential role in lysosome-dependent activation of AMPK and lifespan extension effect by metformin (Chen et al., 2017). However, it is unknown whether AXIN-1 is required for metformin to stabilize STING. Therefore, we generated AXIN-1-deficient H460 cells by CRISPR/Cas9 (*AXIN-1* ^*−/−*^ cells, Figure 4A). In control *AXIN-1* ^*+/+*^ H460 cells, metformin together with activated T cells decreased colony formation of cancer cells. However, in *AXIN-1* ^*−/−*^ cells, this combination had little effect in inhibiting growth of cancer cells (Figure 4B; Supplementary Figure S7A). Similarly, metformin in combination with activated T cells decreased cell viability and inhibited cell proliferation in *AXIN-1* ^*+/+*^ H460 cells, while this effect was abrogated in *AXIN-1* ^*−/−*^ cells (Figures 4C,D; Supplementary Figure S7B). These results indicated specific involvement of AXIN-1 in metformin-enhanced T cell-mediated cancer cell killing. Next, we investigated the contribution of AXIN-1 to stabilization of STING by metformin. Compared to *AXIN-1* ^*+/+*^, *AXIN-1* ^*−/−*^ cells showed decreased STING expression. Moreover, metformin failed to increase STING expression, or to slow down the degradation of STING in *AXIN-1* ^*−/−*^ cells (Figure 4E). To further confirm the role of AXIN-1 in regulation of STING stability, we constructed *AXIN-1* ^*−/−*^ + AXIN1 cells with ectopic expression of AXIN-1 (Supplementary Figure S8A). Although metformin combining activated T cells failed to decrease cell viability and inhibit cell proliferation in *AXIN-1* ^*−/−*^ cells, this effect was rescued in *AXIN-1* ^*−/−*^ + AXIN1 cells (Supplementary Figures S8B, C). Furthermore, metformin increased STING expression and slowed down its degradation in *AXIN-1* ^*−/−*^ + AXIN1 cells (Supplementary Figure S8D). Taken together, *AXIN-1* is required for metformin to stabilize STING and enhance T cell-mediated killing of cancer cells.

### Metformin Enhanced the Co-localization and Binding of AXIN-1 and STING

**FIGURE 5:**
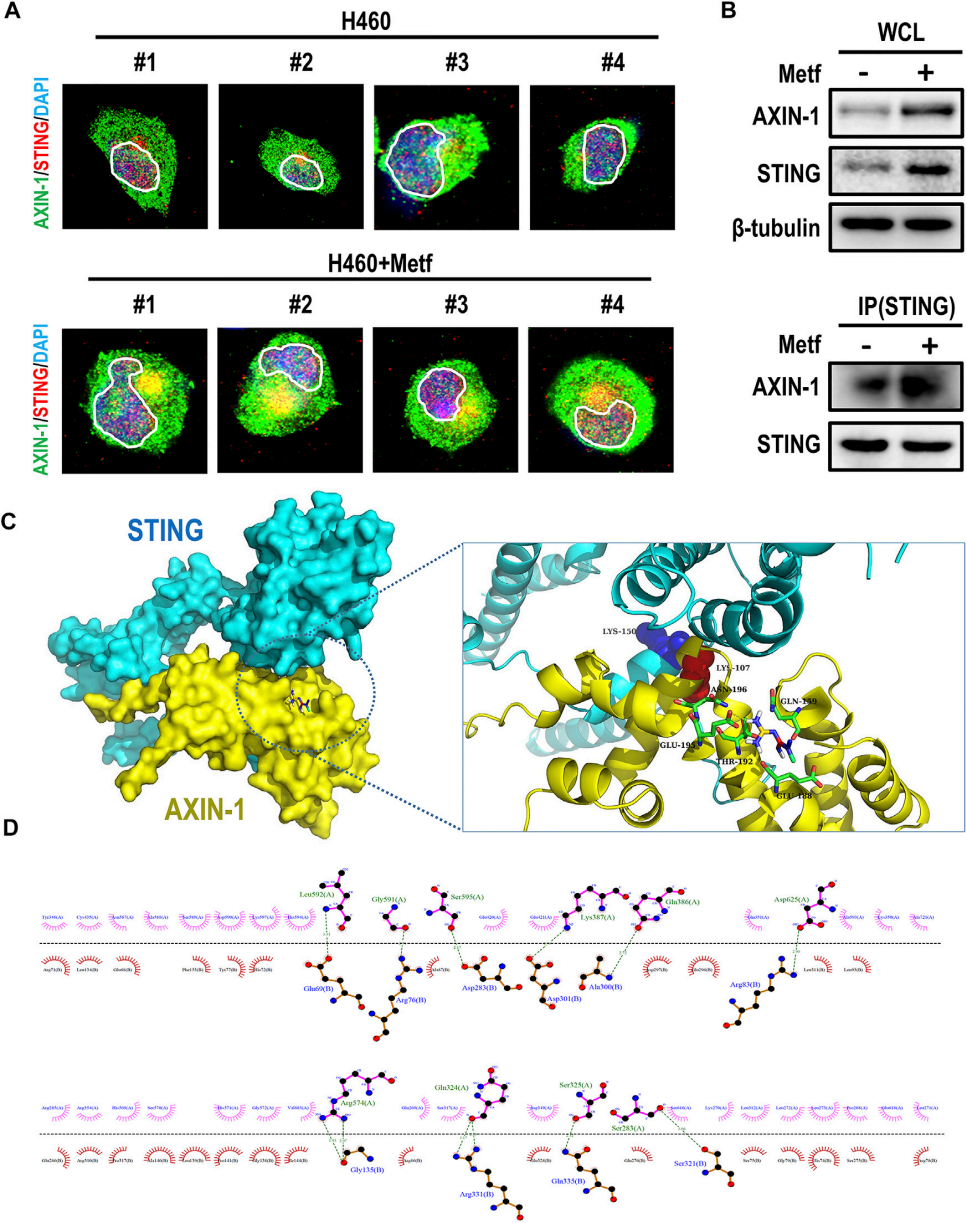
Metformin enhanced the co-localization and binding of AXIN-1 and STING. **(A)**, Representative immunofluorescence images for AXIN-1 and STING of H460 cells following metformin treatment. Yellow signals indicated co-localization. **(B)**, Cell lysates from H460 cells with metformin treatment were immunoprecipitated with an anti-STING antibody and then immunoblotted with anti-AXIN-1 antibody. **(C)**, Protein-protein docking prediction between AXIN-1 and STING. **(D)**, The 2D visualization and interactions between AXIN-1 and STING. The hydrogen bonds and the hydrophobic interactions were shown in green-dashed lines and red arcs, respectively.

To elucidate the mechanism how AXIN-1 stabilizes STING under metformin treatment, we then studied whether AXIN-1 directly interacted with STING. To this end, we performed immunofluorescence staining of AXIN-1 and STING in H460 cells with or without metformin treatment. Compared to the control group, metformin treatment led to obvious co-localization of AXIN-1 and STING (Figure 5A, yellow signal). We then confirmed this finding using immunoprecipitation. As shown in Figure 5B, the binding of AXIN-1 and STING was significantly enhanced under metformin treatment. Next, a protein-protein docking prediction was performed to predict the binding models of AXIN-1 and STING. The docking results suggest that AXIN-1 could bind with STING, and the key amino acid residue Lys150 (K150) in STING was occupied by the loops of the Lys 107 (K107) in AXIN-1 (Figure 5C). On the other hand, the small molecule metformin docks in a pocket of AXIN-1 near the K107 site, which is composed of GLU-195, ASN-196, GLN-159, THR-192, and GLU-188 amino acid residues. Among them, metformin forms hydrogen bonds with GLU-195 and interacts with other amino acid residues (Figure 5C). The interface analysis suggested that interaction between AXIN-1 and STING was maintained by hydrogen bonds and hydrophobic interactions (Figure 5D). Taken together, these results suggest that metformin enhanced the binding of AXIN-1 and STING at K150 site.

### AXIN-1 Promotes Sabilization of STING *via* Competitive Inhibition of RNF5-Mediated K48-Linked Ubiquitination of STING

**FIGURE 6:**
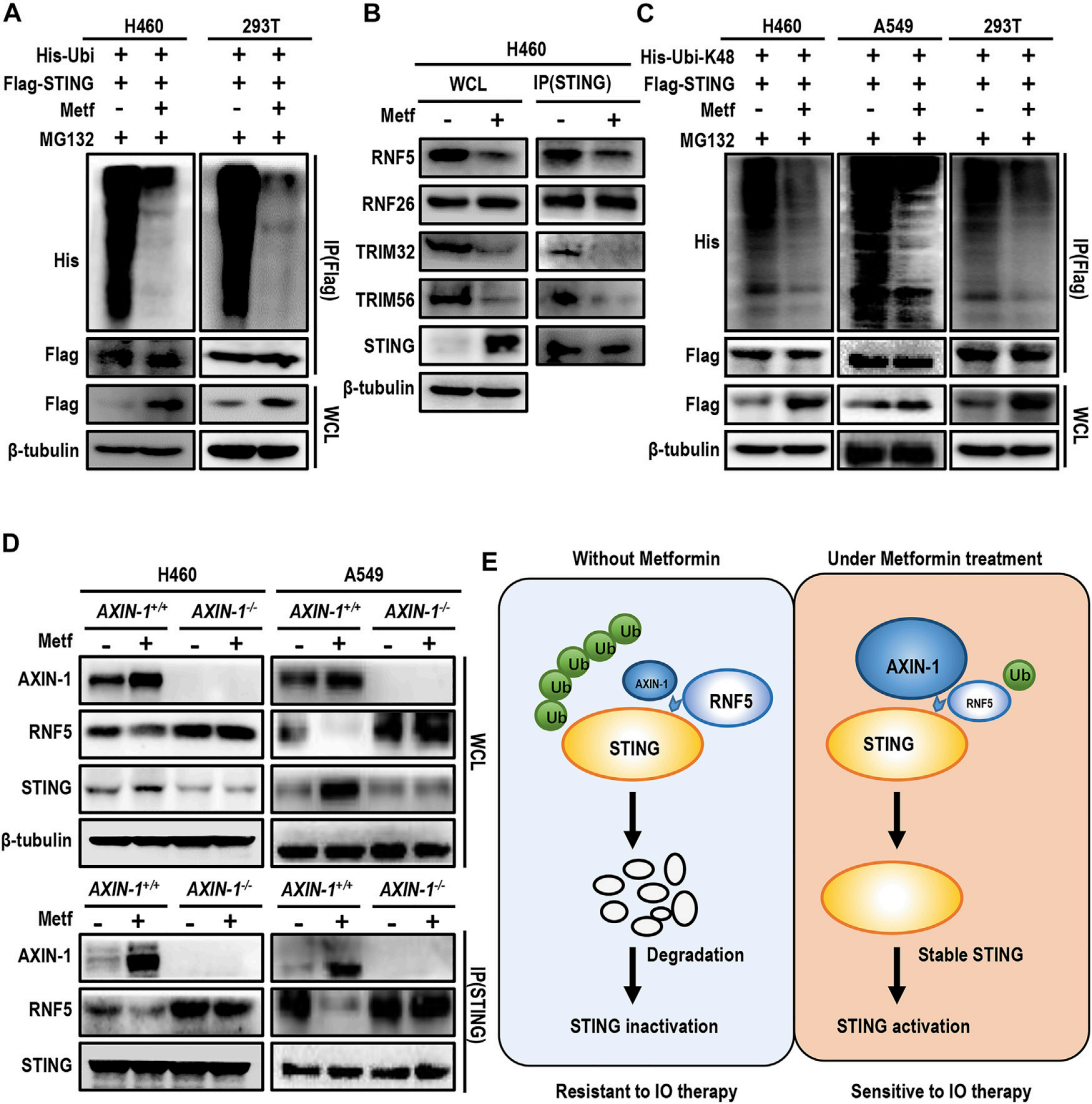
AXIN-1 promotes stabilization of STING via competitive inhibition of RNF5-mediated K48-linked ubiquitination of STING. **(A)**, H460 cells and HEK293T cells were transiently transfected with the indicated plasmids with MG132 for 12 h. Immunoprecipitation analysis of exogenous STING ubiquitination with the indicated antibodies. **(B)**, Cell lysates from indicated groups were immunoprecipitated with an anti-STING antibody and then immunoblotted with the indicated antibodies. **(C)**, Cells were transfected with the indicated plasmids and then immunoprecipitation analysis of K48-linked ubiquitination of exogenous STING was performed. **(D)**, Cell lysates from *AXIN-1* ^*+/+*^ cells or *AXIN-1* ^*−/−*^ cells with different treatments were immunoprecipitated with an anti-STING antibody and then immunoblotted with RNF5. **(E)**, Working model for the mechanism of STING activation by metformin.

STING is reported to be extensively modified by post-translational modification (Chiang and Gack, 2017). For example, ubiquitination of STING at K150 site dynamically regulates the activity of STING. We then asked whether metformin affects ubiquitination of STING. In both H460 cells and 293T cells, metformin treatment significantly decreased STING ubiquitination in the presence of MG132 (Figure 6A). Ubiquitination of STING at K150 site is regulated by several E3 ubiquitin ligases. For example, RNF5 modifies STING at K150 with K48-linked polyubiquitin and promotes STING degradation (Chiang and Gack, 2017). On the contrary, RNF26 catalyzes K11-linked polyubiquitination of STING at K150, and competes with RNF5 to ubiquitinate STING (Fenech et al., 2020). Besides, the E3 ubiquitin ligases TRIM32 and TRIM56 also target STING at K150 for K63-linked ubiquitination, and facilitates the recruitment of TBK1 to STING for STING activation (Yang et al., 2018; Bodda et al., 2020). We then performed immunoprecipitation assay to detect the binding of STING with different E3 ubiquitin ligases under metformin treatment. Results showed that metformin decreased the binding of STING with RNF5, TRIM32, and TRIM56, while had little effect in binding with RNF26 (Figure 6B). Thus, we speculated that metformin decreased STING degradation through inhibition of RNF5-mediated K48-linked ubiquitination. As expected, metformin treatment decreased K48-linked ubiquitination of STING in H460 cells, A549 cells and 293T cells (Figure 6C).

Since AXIN-1 was predicted to bind with STING at K150, we then asked whether AXIN-1 affected the binding of RNF5 and STING. As shown in Figure 6D, metformin significantly enhanced the binding of AXIN-1 and STING, while decreased the binding of STING and RNF5 in both H460 and A549 cell lines. On the contrary, in *AXIN-1* ^*−/−*^ cells, the binding of RNF5 with STING was significantly increased, and metformin treatment failed to decrease the binding of RNF5 with STING. Thus, these results suggest that AXIN-1 competitively inhibited RNF5-STING binding, thus decreasing RNF5-mediated K48-linked ubiquitination of STING (Figure 6E).

## Discussion

In the current study, we reported that metformin enhanced T cell-mediated killing and PD-1 inhibitor efficacy in *STK11* mutant lung cancer. AXIN-1 was required for metformin to stabilize STING via enhanced binding at K150 site and a competitive inhibition of RNF5-mediated K48-linked ubiquitination of STING. Thus, our study indicated that metformin in combination with PD-1 inhibitor may be a potential therapeutic approach for *STK11* mutant lung cancer.

The current study provided a new approach, the combination of metformin and PD-1 inhibitor, to treat *STK11* mutant lung cancer. *STK11* mutation is associated with dismal prognosis in NSCLC. Most *STK11* missense mutations negatively impact upon the LKB1 protein activity (Benci et al., 2016), which then lead to alterations of cancer-associated metabolism, lung cancer initiation, differentiation, local progression and metastatic dissemination (Faubert et al., 2014; Murray et al., 2019). In a large-scale, real-world, retrospective study involving 2407 advanced NSCLC patients, those with *STK11* mutation (13.6% of the whole population) had worse PFS and OS, regardless of treatment types (either chemotherapy or immunotherapy) or treatment lines (either first- or second-line settings) (Shire et al., 2020). Besides, *STK11* mutation was identified as a major driver of primary resistance to PD-1/PD-L1 blockade in NSCLC (Skoulidis et al., 2018). In a retrospective analysis, advanced non-squamous NSCLC patients with *STK11* mutation did not achieve clinical benefit from pembrolizumab plus chemotherapy (Skoulidis et al., 2019). Currently, very few therapeutic interventions have been developed to specifically treat *STK11* mutant tumors. In the current study, we found that metformin enhanced T cell-mediated killing of lung cancer cells *in vitro* through colony formation assay, cell viability CCK-8 assay and Ki67 incorporation assay. Moreover, we found that metformin in combination with PD-1 inhibitor enhanced anti-tumor efficacy *in vitro* and *in vivo*. In the future, *in vivo* rescue experiments are needed to demonstrate whether metformin can enhance the efficacy of PD-1 inhibitor. Previously, metformin has also been shown to induce immune cell infiltration in tumors, and promote antitumor CD8^+^ T cell immune responses (Cha et al., 2018). These reports, together with our findings, suggest that metformin has the promising potential to overcome the primary resistance to ICIs in *STK11* mutant lung cancer.

Although metformin has been identified as a potentially efficacious antitumor agent, results from clinical studies are not always satisfactory. We previously reported that in EGFR mutant NSCLC without diabetes, metformin in combination with gefitinib resulted in non-significantly worse outcomes but increased the risks of diarrhea (Li et al., 2019c). Recently, in a randomized trial involving unresected locally advanced NSCLC without diabetes, the addition of metformin to chemoradiotherapy was associated with worse treatment efficacy and increased toxic effects compared with chemoradiotherapy alone (Tsakiridis et al., 2021). In another randomized phase II study, addition of metformin to chemotherapy provided no survival benefit in unselected NSCLC patients, while it significantly improved the survival of the selected squamous cell carcinoma patients with high FDG uptake (Lee et al., 2021). These studies indicate that only specific NSCLC subgroups may benefit from adding metformin to standard therapy. Careful patient selection on biomarker expression will be essential to select suitable population who may benefit from metformin. Recent studies found that *STK11* mutation exposed cancer cells to metabolic crisis and apoptosis, and sensitized those cells to the antidiabetic compounds (Shackelford et al., 2013), which lower intracellular ATP levels by inhibiting mitochondrial oxidative phosphorylation. Metformin could prevent acquired resistance to cisplatin in *STK11* mutant lung cancer through reducing the number of tumor-initiating cells (Moro et al., 2018). These results, together with findings from the current study, suggest that *STK11* mutant lung cancer may be the potential population that can benefit from adjuvant metformin. Currently, a phase II study is being conducted to exploit metformin and fasting-mimicking diet to improve the efficacy of chemotherapy in advanced *STK11* mutant lung adenocarcinoma (Vernieri et al., 2019).

There is a compelling need to understand the resistance mechanism to PD-1 inhibitors in *STK11* mutant NSCLC, so as to envisage effective therapeutic interventions. *STK11* mutation was associated with a specific “cold” tumor immune microenvironment (TIME), characterized by production of pro-inflammatory cytokines, decrease in tumor-infiltrating CD8^+^ lymphocytes and low PD-L1 expression on tumor cells (Koyama et al., 2016). Recent studies suggest that STING may play an important role in “cold” to “hot” transformation of TIME (Della Corte and Byers, 2019). STING is a signaling molecule that controls the transcription of many host defense genes, including pro-inflammatory cytokines and chemokines, and type I IFNs (Ishikawa and Barber, 2008; Ishikawa et al., 2009). Activation of STING pathway promotes the trafficking and infiltration of T cells to tumors, and is required for the recognition and killing of cancer cells by T cells (Zhu et al., 2019). Ectopic expression of STING in *STK11* mutant cells engages IRF3 and STAT1 signaling, leading to the release of the immune inflammatory cytokines IFNβ, CXCL10, CCL5, GM-CSF, CCL3, and IL1α, and suppression of IL6 (Kitajima et al., 2019). In the current study, we found that metformin can enhance STING stabilization and activation *in vitro*, and increased STING expression *in vivo* in PBMCs-CDX model. Moreover, STING was required for metformin to enhance T cell-mediated killing of cancer cells. In a previous report, metformin was also reported to activate STING/IRF3/IFN-β pathway and promote T cells infiltration, through inhibition of AKT phosphorylation (Ren et al., 2020). Taken together, these results suggest that metformin enhances PD-1 inhibitor efficacy in *STK11* mutant NSCLC through activation of STING.

Preclinically and clinically, STING agonists have been investigated for cancer immunotherapy. Cyclic dinucleotides (CDNs), or CDN derivatives are a representative class of STING agonists that can elicit immune responses. MK-1454, a synthetic CDN which binds directly to STING, showed a safe tolerability profile and encouraging efficacy together with pembrolizumab in a phase I trial (NCT03010176) (Harrington et al., 2018). However, natural CDNs are hydrophilic, with negative charges and are susceptible to enzymatic degradation, and requires intratumor injection, leading to low bioavailability in target tissues and challenges in clinical practice in patients whose tumors are not easily accessible, such as lung cancer (Su et al., 2019). Recently, it was reported that an intravenous STING agonist, which was made up of two linked amidobenzimidazole compounds, showed strong antitumor activity in a colon cancer model (Ramanjulu et al., 2018). The current study found that metformin may be potentially applied as an activator of STING to activate STING and its downstream pathway, thus enhancing anti-cancer immune responses. Given a satisfactory safety profile, metformin holds the potential to be applied in combination with ICIs to activate STING and enhance anti-cancer immune response.

In search of mechanism how metformin activates STING signaling, we found that AXIN-1 was required for stabilization of STING by metformin. AXIN-1 is a scaffold protein which serves as a platform for functioning of multiple proteins. Previously, it was reported that AMPK activation by metformin was dependent on the formation of the v-ATPase-Ragulator-AXIN/LKB1-AMPK complex (Zhang et al., 2016; Chen et al., 2017). In the current study, we found that AXIN-1 was required for metformin to enhance T cell-mediated killing of cancer cells. Also, knockout of AXIN-1 abolished the effect of metformin to stabilize STING. As previously reported, AXIN-1 was required for the degradation complex of β-catenin, and served as a negative regulator of Wnt/β-catenin signaling (Jackson et al., 2020). In the current study, we found that in *STK11* mutant lung cancer without LKB1 expression, AXIN-1 served as a platform for binding with STING, which was enhanced by metformin treatment. The binding of AXIN-1 with STING reduced RNF5-mediated K48-linked ubiquitination of STING. Therefore, AXIN-1-based STING stabilization was required for metformin to activate STING pathway and enhance immunotherapy efficacy in *STK11* mutant lung cancer.

In conclusion, the current study demonstrated that metformin overcomes primary resistance to PD-1 inhibitor in *STK11* mutant lung cancer. Future clinical studies are required to further investigate the clinical benefits of metformin combined with PD-1 inhibitors in *STK11* mutant lung cancer.

## Data Availability

The original contributions presented in the study are included in the article/Supplementary Material, further inquiries can be directed to the corresponding authors.
