# Supplementary material for: Metformin Combining PD-1 Inhibitor Enhanced Anti-Tumor Efficacy in STK11 Mutant Lung Cancer Through AXIN-1-Dependent Inhibition of STING Ubiquitination
Source: Front Mol Biosci. 2022 Feb 23;9:780200. doi: 10.3389/fmolb.2022.780200 (PMC8905189; doi:10.3389/fmolb.2022.780200)
Supplement: Supplementary file 1 [file DataSheet1.PDF]

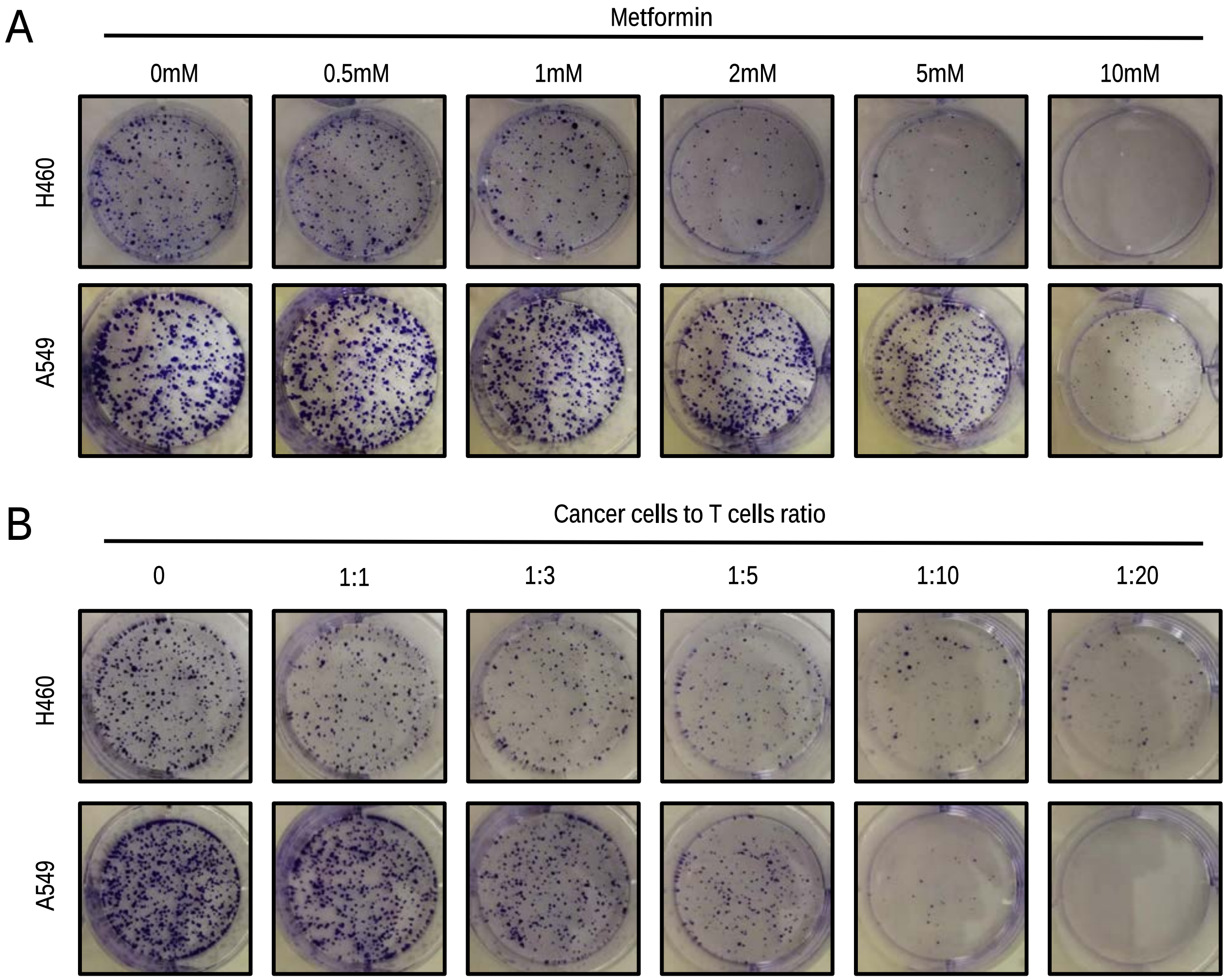

Supplemental Figure 1. Effect of metformin or T cells alone on growth of H460 and A549 cells. A, H460 cells and A549 cells were cultured with metformin of different doses (0.5 mM to 10 mM as indicated) for 48h. B, H460 cells and A549 cells were co-cultured with activated T cells (cancer cells to T cells ratio, 1:1 to 1:20 as indicated) for 48 h.

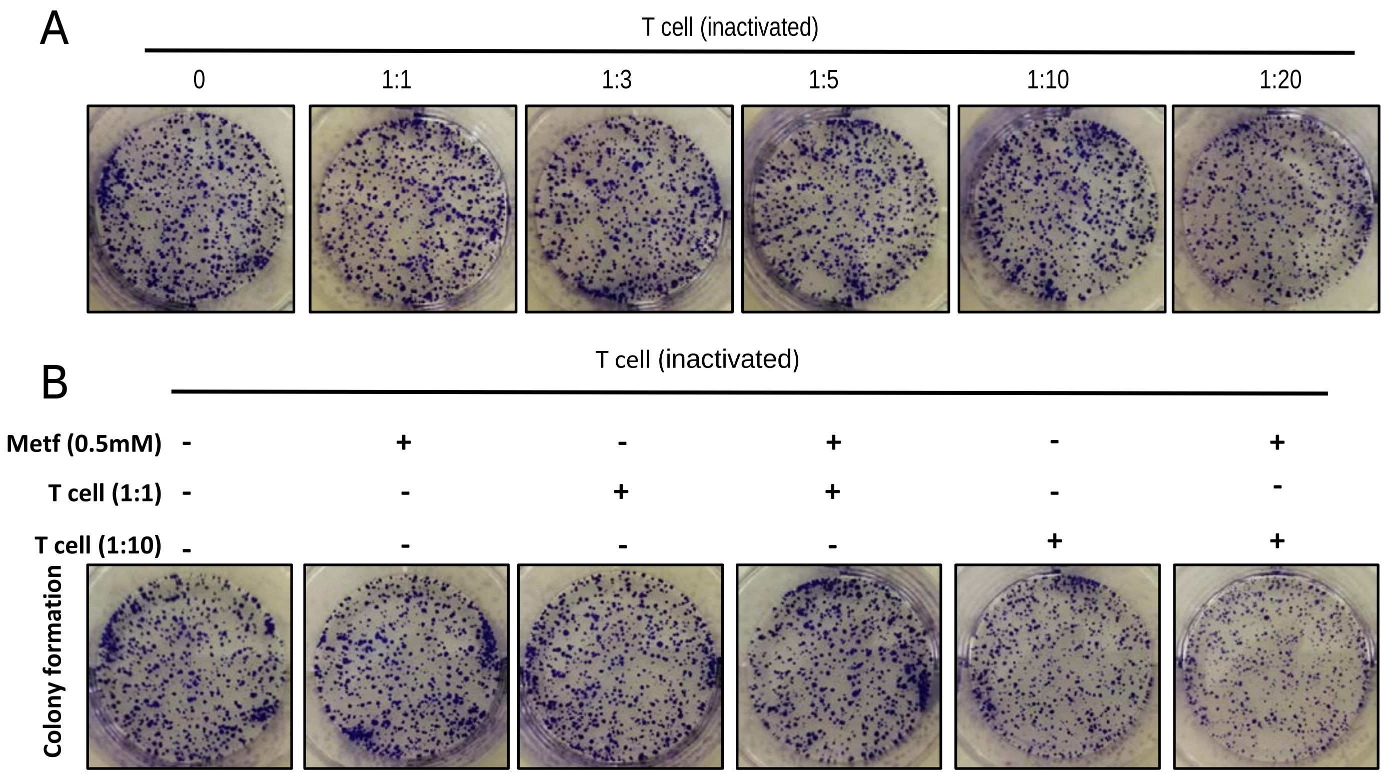

Supplemental Figure 2. **metformin failed to enhance T-cell mediated killing of H460 cells in inactivated T cells**.(A) H460 cells were incubated with inactivated T cells (1:1, 1:3, 1:5, 1:10 and1:20), and then colony formation assay were performed. Inactivated T cells show little effect on tumor growth .(B) H460 cells were incubated with inactivated T cells (1:1 or 1:10) in combination with Metformin (0.5mM) and then colony formation analysis was performed.

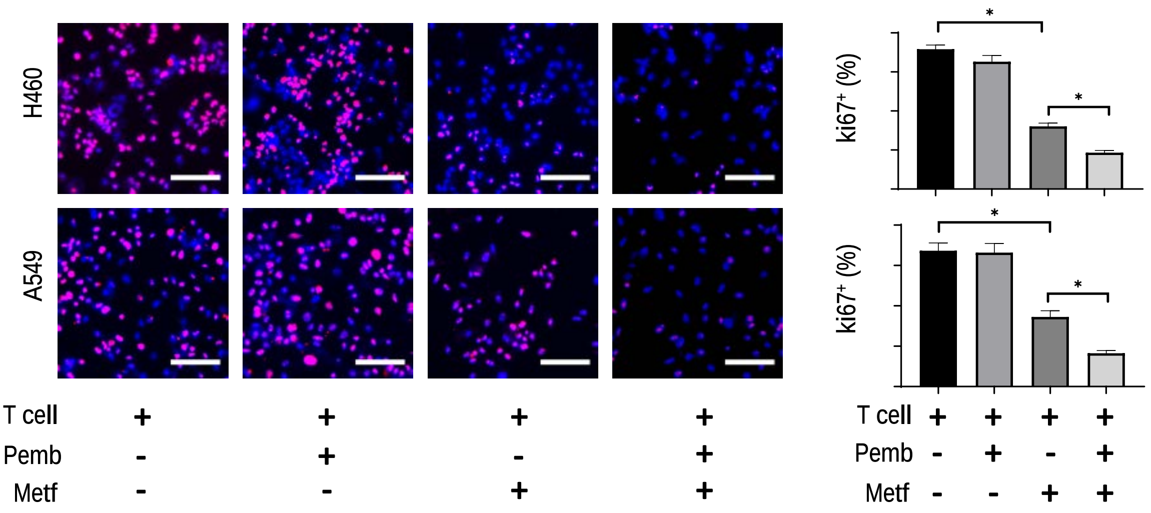

Supplemental Figure 3. Metformin enhanced the anti-tumor efficacy with PD-1 inhibitor in vitro. Ki67 incorporation assay werand A549 cells treated as indicated. Activated T cells (1:1 ratio to cancer cells), metformin (0.5mM) or pembrolizumab (10ug/ml) were added to the culture medium for 48 h. Cells were then counterstained with DAPI. Data represent mean± SEM. *, $p<0.01$. Scale bars: 100 μm.

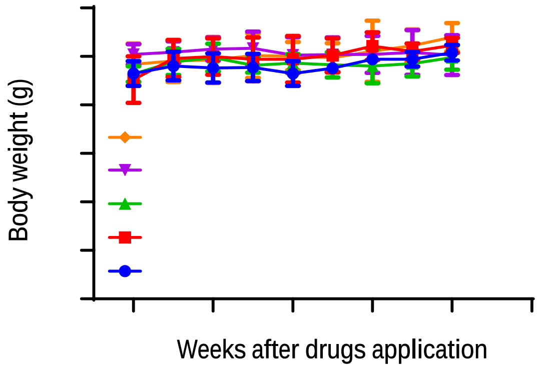

Supplemental Figure 4. Mice weight of each group**.** Body weight of each mice among different groups was presented as mean ± SEM (n = 5).

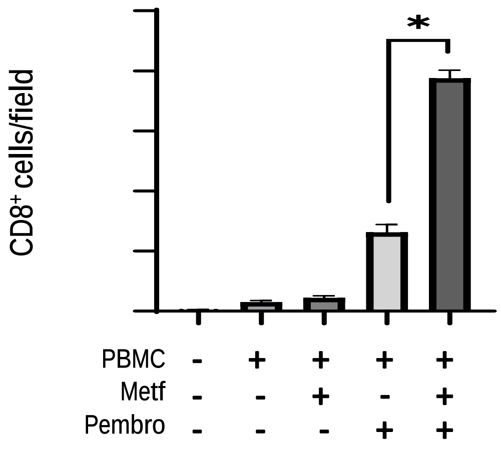

Supplemental Figure 5 The numbers of stained CD8+ T cells per field in different treatment groups were presented as mean± SEM.*, p<0.01.

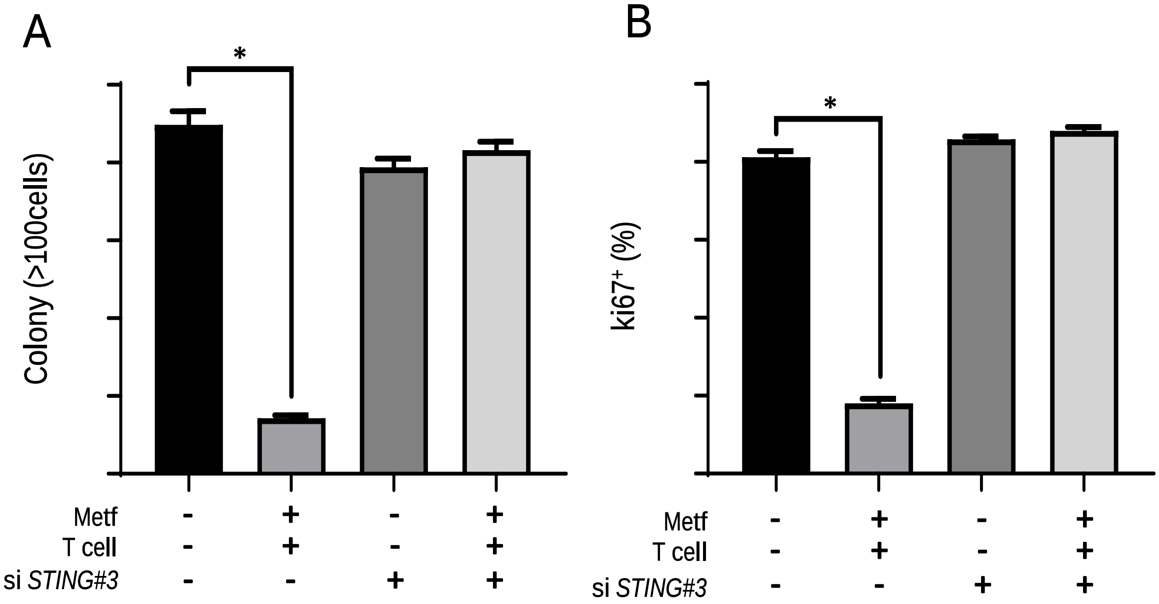

Supplemental Figure 6 Cell colonies and the percentage of Ki67-positive cells among indicated groups were presented as mean ± SEM. *, p<0.01.

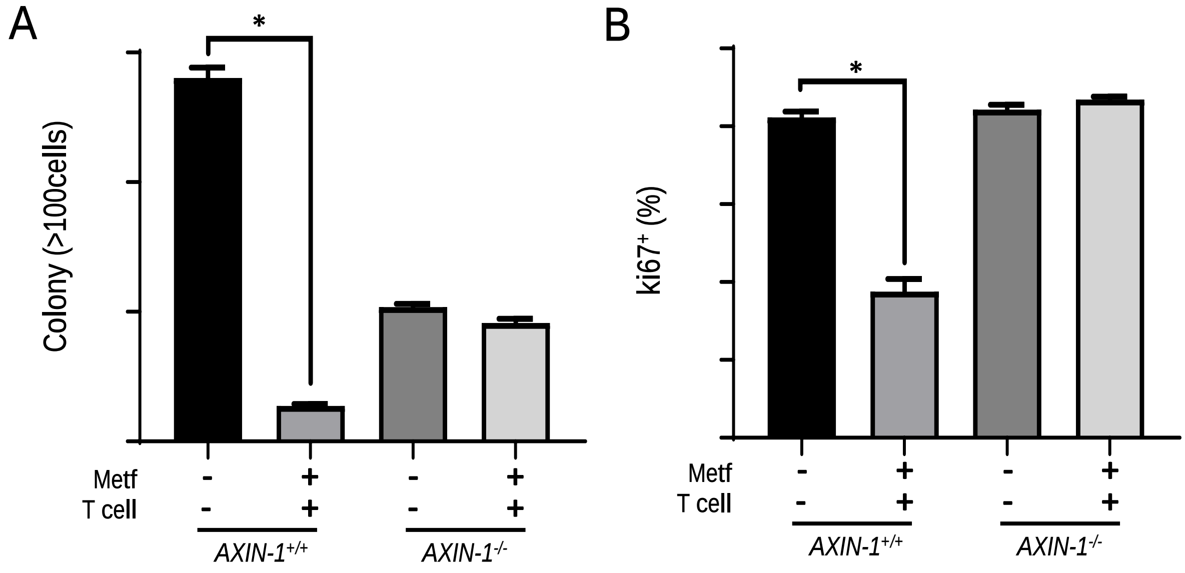

Supplemental Figure 7 Cell colonies and the percentage of Ki67-positive cells among indicated groups were presented as mean ± SEM. *, $p<0.01$.

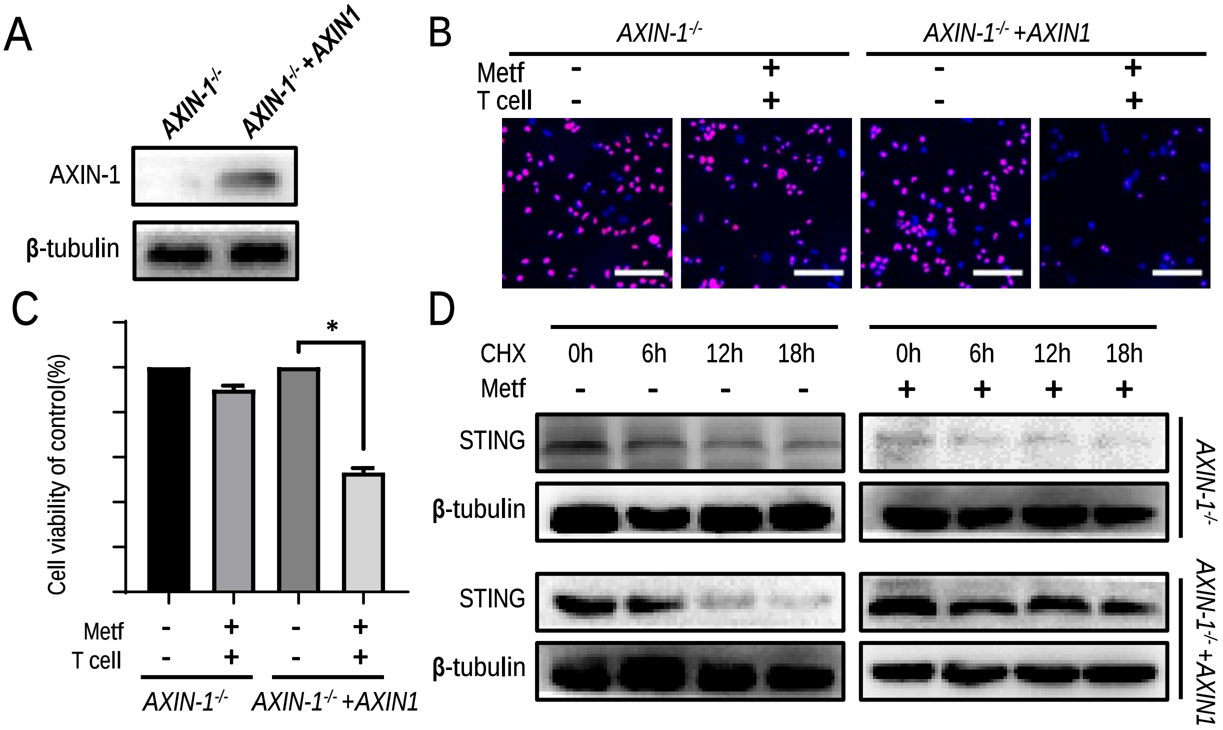

Supplemental Figure 8. Rescue experiment to confirm the role of AXIN-1 for metformin to stabilize STING. A, Cell lysates from *AXIN-1*$^{-/-}$ cells and *AXIN-1*$^{-/-}$ +AXIN1 cells were subjected to western blot. B, Ki67 incorporation assay on *AXIN-1*$^{-/-}$ cells and *AXIN-1*$^{-/-}$ +AXIN1 cells treated as indicated. Cells were counterstained with DAPI. C, Cell viability CCK-8 assay for *AXIN-1*$^{-/-}$ cells and *AXIN-1*$^{-/-}$ +AXIN1 cells treated as indicated. Data are shown as mean ± SEM. *, p<0.001. D, *AXIN-1*$^{-/-}$ cells and *AXIN-1*$^{-/-}$ +AXIN1 cells were treated with 10 Mm CHX at indicated intervals in the presence of metformin or not. *AXIN-1*$^{-/-}$ **+AXIN1:** construction of AXIN1 expression in *AXIN-1*$^{-/-}$ cells.
